# Tolerability of intensified intravenous interferon alfa-2b versus the ECOG 1684 schedule as adjuvant therapy for stage III melanoma: a randomized phase III Italian Melanoma Inter-group trial (IMI – Mel.A.) [ISRCTN75125874]

**DOI:** 10.1186/1471-2407-6-44

**Published:** 2006-02-27

**Authors:** Vanna Chiarion-Sileni, Paola Del Bianco, Antonella Romanini, Michele Guida, Adriano Paccagnella, Maurizio Dalla Palma, Emanuele Naglieri, Ruggero Ridolfi, Barbara Silvestri, Maria Michiara, Gian Luca De Salvo

**Affiliations:** 1 Department of Medical Oncology, University Hospital, Padua, Italy; 2 Clinical Trials and Biostatistics Unit, Istituto Oncologico Veneto, Padua, Italy; 3 Medical Oncology Unit, S. Chiara Hospital, Pisa, Italy; 4 Medical Oncology Unit, Ospedale Oncologico, Bari, Italy; 5 Medical Oncology Unit, SS Giovanni e Paolo Hospital, Venezia, Italy; 6 Medical Oncology Unit, S. Bortolo Hospital, Vicenza, Italy; 7 Oncology Dept – Pierantoni Hospital- Forlì, Italy; 8 Medical Oncology Unit, General Hospital, Noale (Ve), Italy; 9 Medical Oncology Unit, General Hospital, Parma, Italy

## Abstract

**Background:**

High-dose interferon alfa-2b (IFNalfa-2b), according to the ECOG 1684 schedule, is the only approved adjuvant treatment for stage III melanoma patients by the FDA and EMEA. However, the risk/benefit profile has been questioned limiting its world-wide use. In the late nineties, the Italian Melanoma Inter-group started a spontaneous randomized clinical trial (RCT) to verify if a more intense, but shorter than the ECOG 1684 regimen, could improve survival without increasing the toxicity profile. The safety analysis in the first 169 patients who completed the treatment is here described.

**Methods:**

Stage III melanoma patients were randomized to receive IFNalfa-2b 20 MU/m^2^/d intravenously (IV) 5 days/week × 4 weeks, repeated for three times on weeks 9 to 12, 17 to 20, 25 to 28 (Dose-Dense/Dose-Intense, DD/DI, arm), or IFNalfa-2b 20 MU/m^2^/d IV 5 days/week × 4 weeks followed by 10 MU/m^2 ^subcutaneously (SC) three times per week × 48 weeks (High Dose Interferon, HDI, arm). Toxicity was recorded and graded, according to the WHO criteria, as the worst grade that occurred during each cycle.

**Results:**

The most common toxicities in both arms were flu-like and gastrointestinal symptoms, leukopenia, liver and neuro-psichiatric morbidities; with regard to severe toxicity, only leukopenia was statistically more frequent in DD/DI arm than in HDI arm (24% vs 9%) (p = 0.0074), yet, this did not cause an increase in the infection risk. Discontinuation of treatment, due to toxicity, was observed in 13 and 17% of the patients in the DD/DI and HDI arm, respectively. The median actual dose intensity delivered in the DD/DI arm (36.4 MU/m^2^/week) was statistically higher than that delivered in the HDI arm (30.7 MU/m^2^/week) (p = 0.003).

**Conclusion:**

Four cycles of intravenous high-dose IFNalfa-2b can be safely delivered with an increase in the median dose intensity. Efficacy results from this trial are eagerly awaited.

## Background

Melanoma is the most aggressive skin cancer and poses an increasingly important health problem. Surgery offers the best chance for cure in the early stage of disease. However, when melanoma is detected with a thickness of > 4.0 mm or in the presence of regional lymph node involvement, the risk of recurrence and death becomes higher. The 5-year overall survival, in node positive patients, ranges from 26 to 69%, depending on the number of lymph nodes involved, whether they were identified microscopically or clinically, or whether the primary melanoma was ulcerated or not [1,2].

Moreover, chemotherapy and immunotherapy have a limited efficacy when the disease becomes metastatic, and in those patients the median survival time is estimated to be 8.1 months (95% confidence interval: 7.3–8.9), with a long-term survival over 5 years of 2.3% [3]. Therefore, it is of paramount importance to find effective adjuvant therapies for melanoma patients at high risk of recurrence.

Interferon-α2b (IFNα2b) has proved to have some consistent anti tumor activity in locally advanced [4] and metastatic disease and has been tested in clinical trials, in the adjuvant setting, in a variety of dosage regimens. So far, in patients with node involvement and/or a Breslow's thickness > 4 mm (stage IIB and III), only 1-year of high dose IFNα2b (HDI) has been shown to significantly reduce the risk of relapse and death compared with observation [5] and with a ganglioside vaccine [6]. However, this regimen is associated with significant toxicity and, despite its approval by the US Food and Drug Administration and by the European Medicines Agency, its widespread acceptance has been limited: it is estimated that less than 20% of the Italian node-positive melanoma patients receive adjuvant IFNα2b treatment. Concerns about its tolerance increased after the publication of the E1690/S9111/C9190 inter-group study which confirmed the benefits on disease-free survival, as seen in the ECOG 1684 study, but failed to show any survival benefit, thus increasing the skepticism about the efficacy of this treatment. The subsequent E1694/S9512/C509801 inter-group study showed a significant increase in disease-free and overall survival of patients treated with HDI compared with the GM2/KLH/QS21 ganglioside vaccine. However, this finding did not change the world-wide debate on whether the clinical benefit (25% improvement in the relative overall survival) [7] could be offset by its toxicity. This issue has not been resolved yet, despite the study of Cole et al [8] showed a gain in the quality-of-life-adjusted survival time in the treated group when compared to the observation group.

The distinguishing feature of the ECOG 1684 regimen was a 4 week, intravenous, high dose induction phase, followed by 48 weeks of subcutaneous administration. The analysis of the survival curves in the E1684 study points out an early separation in the curves, thus suggesting that the higher plasma concentrations, achieved with the intravenous induction phase, may be critical and necessary for clinical benefit [5]. With the aim of further exploring this aspect, and of reducing the duration of the treatment, the Italian Melanoma Inter-group planned a spontaneous randomized study to determine if the intensive intravenous induction regimen repeated for 4 times could improve relapse-free and overall survival compared with the standard 1-year HDI regimen.

We chose to give four induction cycles in order to administer a similar amount of total interferon in the two treatment arms, and simultaneously to reduce the time spent by the patients on therapy. We decided to restrict the accrual only to node-positive melanoma patients in order to have a more homogeneous risk population. Acknowledging that the toxicity of HDI is a substantial issue, we planned to carry out an interim safety analysis once at least 50% of all patients had completed their treatment. We hereby report the results of this safety analysis in the first 169 patients treated.

## Methods

### Patient selection

Eligible patients had a histologically-proven stage III primary or recurrent melanoma of cutaneous origin, or clinically detected nodal metastasis arising from an unknown primary, without evidence of systemic disease (N1a, N1b, N2a, N2b, N3, according to the revised AJCC stage groupings for melanoma [9]). Patients with satellite or in-transit metastases and patients with extra capsular nodal involvement or recurrence after a previous lymph node dissection, were excluded from the study. Patients had to undergo radical excision of primary tumor with at least 2 cm margins and be submitted to therapeutic lymphadenectomy within 60 days before randomization. Patients with more than one lymphatic basin drainage involved could be randomized providing all the lymphatic basins had been radically resected.

The definition of nodal metastasis required the identification of tumor cells by routine stains, and neither immunohistochemical stains nor positive reverse transcriptase polymerase chain reactions alone were considered sufficient for study entry.

Patients were required to meet the following criteria: an age of 18 years or older, ECOG performance status of 0–1, adequate organ function (bilirubin, SGOT, serum creatinine, and BUN within normal ranges) and no significant medical or psychiatric or autoimmune co-morbidity.

Exclusion criteria were an age of more than 70 years, pregnancy, or lactation, and previous adjuvant therapy.

Randomization was carried out by telephone through the Clinical Trials and Biostatistics Unit, the study data center responsible for data management. A system of random permuted blocks within the participating center strata was used. Data were collected on paper case report forms and recorded on an electronic database designed specifically for the management of the trial.

All patients gave their written informed consent to receive the treatment. The study was approved by the Ethics Committee of each participating center, in compliance with the Helsinki Declaration.

### Treatment

All patients were randomly assigned to the two treatment groups: IFNα2b 20 MU/m^2 ^/d IV 5 days/week × 4 weeks, repeated for three times on weeks 9 to 12, 17 to 20, 25 to 28 (Dose-Dense/Dose-Intense, DD/DI, arm), or IFNα2b 20 MU/m^2 ^/d IV 5 days/week × 4 weeks followed by 10 MU/m^2 ^SC three times per week × 48 weeks (High Dose Interferon, HDI, arm). The IV IFNα2b doses were administered in 20' diluted in 100 ml of saline, the subcutaneous doses were self-administered. We defined 4 weeks of treatment as a cycle, in both arms, in order to evaluate the dose intensity and the dose delivered. Patients received oral paracetamol 1,000 mg 30' before IFNα2b administration, and subsequently, as needed, up to a maximum of 3,000 mg/d, to prevent and control IFN-related fever and flu-like symptoms; ondansetron 8 mg or granisetron 3 mg IV were used to prevent nausea and emesis, omeprazole 20 or 40 mg/d was used in case of gastric acidosis or in patients with a history of gastric ulcer disease; multivitamin intake and physical exercise were strongly recommended to all patients, and iron supplement was added when the hemoglobin decreased more than three mg from the initial value. Patients were monitored for toxicity and compliance, by physical examination, hematology and serum chemistry profiles, weekly during the intravenous administration of IFNα2b and monthly during the subcutaneous administration, then, at the end of the treatment, every 3 months for a period of 2 to 5 years, and after the fifth year, every 6 months for disease progression.

The toxicity was recorded and graded, according to the World Health Organization (WHO) criteria, as the worst grade that occurred during each cycle. Treatment was resumed with a 30% dose reduction in case of a grade 3/4 toxicity; after a second episode of grade 3/4 toxicity, a 60% dose reduction was required. In case of complete recovery during the rest period, a dose re-escalation was attempted at subsequent cycles, for patients randomized to DD/DI arm. Dose re-escalation was not attempted otherwise.

### Statistical methods

The primary endpoint of the study was the 5-year overall survival in the intent-to-treat population. Secondary endpoints were relapse-free survival, site of relapse, safety and prospective evaluation of quality of life.

The sample size was determined by assuming a 55% 5-year overall survival for patients treated in the HDI arm and hypothesizing an increase of 15% in patients treated in the DD/DI arm; a recruitment of at least 328 patients was required to verify the hypothesis with an 80% power and a 5% error (2-sided test). An interim analysis was planned when at least 50% of the required patients had completed the planned treatment, to verify whether or not it was feasible to administer high doses of Interferon only intravenously without significantly increasing the toxicity. No formal criteria for study discontinuation were defined but the decision to continue or discontinue was left up to the protocol committee board.

Toxicity was analyzed in an "as treated" population, provided they had received at least one dose of therapy. To assess toxicity and feasibility of the treatment, the two arms were compared in terms of all grade distribution using Wilcoxon' s rank sum test. Difference in occurrence of Grade 3/4 toxicity events between arms was assessed using Fishers exact test.

Actual Dose Intensity (ADI) of Interferon was calculated as recommended by Hryniuk [10], the number of IFNα2b MU being delivered per square meter per week for each patient during the whole treatment. The median ADI was compared using Wilcoxon' s rank sum test.

Adjustments for multiplicity were not made. All p values were based on a two-sided testing, and statistical analyses were carried out with SAS statistical software (Release 8.02; SAS Institute, Cary, NC).

## Results

### Patient characteristics

From November '98 to July '05, 208 patients were enrolled in the still ongoing study. This safety report considered only the first 169 patients enrolled until December 03, who had had enough time to complete the treatment ("safety evaluable population"). Eighty-eight patients were enrolled in the DD/DI arm and 81 in the HDI arm. Three patients, two in DD/DI arm and one in HDI arm respectively, did not start the treatment, one because of lung metastases, whereas the other two withdrew their consent immediately after the randomization.

Table 1 shows the demographic and baseline disease characteristics of the patients. There were 101 males and 65 females, with a mean age of 49.1 years (SD 13.3). More than 94% of patients had ECOG Performance Status equal to 0. Ninety patients (54%) had the primary melanoma located on the trunk, and in 40% of patients the melanoma was ulcerated. The largest subgroup of patients (61%) had one involved lymph node, 26% had two to three involved lymph nodes, and 11% had four or more involved lymph nodes. The main prognostic characteristics were balanced between the treatment groups.

### Toxicity evaluation

Adverse events per WHO grade of DD/DI arm vs. HDI arm are reported in Table 2. Flu-like symptoms, (fatigue, fever and arthro-myalgia), nausea/vomiting, anorexia, cytopenia, elevation of liver enzymes, and neuro-psychiatric symptoms were the most frequently noted events in both arms. The distribution of all grades of toxicity was similar in the two groups, except for leukopenia, which was higher in DD/DI arm (p = 0.016). We also observed a statistically significant higher frequency of hypotension in HDI arm compared to DD/DI arm, however only grades 1 and 2 were observed.

Considering severe toxicity (grades 3 and 4), fatigue and fever, leukopenia and granulocytopenia, elevation of liver enzymes and mood alteration were the most common side effects reported. The only statistically significant difference was related to leukopenia, which was more frequent in the DD/DI arm than the HDI arm (24.4% vs. 8.7% respectively, p = 0.0074); yet, this increase was not associated to a higher risk of infectious disease.

**Figure 1:**
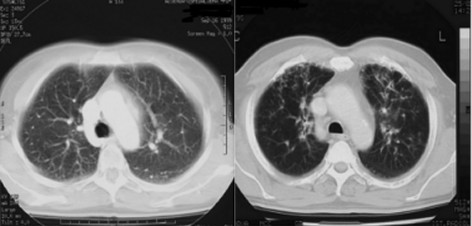
**Computed tomography scan during the interferon treatment. **Computed tomography scan, performed after the 20^th ^week for DD/DI patient (left) and during the 52^nd ^week for HDI patient (right), confirmed mild ground-glass opacities in both lungs with bronchial wall and interstitial thickening. Sputum cultures were negative for bacteria, fungi, and acid-fast bacilli.

**Figure 2:**
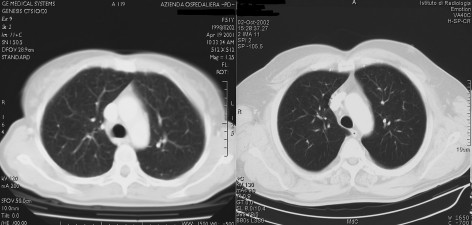
**Computed tomography scan after the end of the interferon treatment. **Computed tomography scan for DD/DI patient (left) and HDI patient (right) performed after the end of treatment showed the disappearance of radiographic infiltrates.

Two patients, one in DD/DI arm and one in HDI arm, suffered from a significant pulmonary toxicity, clinically characterized by a short history of progressive exertional dyspnea associated with dry cough, muscular pain and fever. The patient in DD/DI arm became symptomatic after the 20^th ^week of intravenous IFNα2b administration (14 MU/m^2^), the patient in HDI arm became symptomatic during the 52^nd ^week of subcutaneous IFNα2b administration (8 MU/m^2^). On auscultation, bibasilar coarse crackles were present, chest X-ray revealed patchy bilateral peripheral infiltrates and reticulonodular opacities. A computed tomography scan (Fig. 1) confirmed mild ground-glass opacities in both lungs with bronchial wall and interstitial thickening. Sputum cultures were negative for bacteria, fungi, and acid-fast bacilli. An IFN-induced interstitial pneumonitis was suspected, and both patients were treated with clorfenamine 16 mg/day, and acetil cysteine, avoiding the use of corticosteroids. We decided to continue the treatment in the patient in DD/DI arm at a lower dose of 8 MU/m^2^. Despite an improvement in dyspnea and cough, radiographic infiltrates disappeared only after the end of treatment (Fig. 2).

### Delivered treatment and dose intensity

Overall, 973 cycles were delivered, 272 in DD/DI arm and 701 in HDI arm. Dose modification was necessary in 200 (73.5%) cycles in DD/DI arm and in 514 (73.3%) cycles in HDI arm, respectively, and this dose reduction was higher than 20% in 136 cycles (68%) in DD/DI arm and in 338 (65.8%) in HDI arm. (Table 3).

As shown in table 4, fifty-four patients (62.8%) received all cycles of DD/DI regimen and 32 patients (40.0%) completed the HDI regimen. Toxicity was the cause for treatment discontinuation in 11 patients (12.8%) in DD/DI arm and 14 (17.5%) in HDI arm. Patient's refusal, often observed in association with mood alterations (e.g. minor depression), and not connected to laboratory abnormalities, was reported in the 12.8% of patients in DD/DI arm and 18.7% in HDI arm. Disease progression was the reason for treatment discontinuation in 29 patients (DD/DI + HDI arm, 17.5%).

The fraction of patients able to tolerate ≥ 80% of the target scheduled dosage of IFN therapy at four points in time is listed in table 5. At 28 weeks, 36.4% of patients in the DD/DI arm and 43.4% of patients in the HDI arm had received ≥ 80% of the planned dose.

The planned dose intensity was 50 MU/m^2^/week for the DD/DI arm and 35.4 MU/m^2^/week for the HDI arm. The median actual DI was 36.4 MU/m^2^/week for patients in the DD/DI arm and 30.7 MU/m^2^/week in the HDI arm, respectively. (Table 6) The reduction of actual DI with respect to the planned DI was greater in DD/DI arm than in HDI arm, however the administered DI remained significantly higher in DD/DI arm (p = 0. 003), irrespectively to the dose intensity reduction.

### Supportive therapy

Supportive therapy is described separately in the first cycle, similarly for both arms, and subsequent cycles, because of the different treatment delivery profile (day-hospital for DD/DI arm and outpatients for the maintenance phase of HDI arm). As expected, supportive therapy was similar in the first cycle. During the subsequent cycles of treatment, anti-emetics were used more frequently in DD/DI arm (62.0% vs. 30.4%, respectively, Table 7).

## Discussion and conclusion

The present study was undertaken to investigate whether the risk/benefit ratio of traditional HDI, as introduced by the ECOG group, could be improved by increasing the activity of HDI by administering the drug in a dose-dense/dose-intense (DD/DI) fashion, while maintaining the known toxicity profile.

Toxic side effects, uncertainties about its efficacy, its relative economic burden, and burdensome treatment duration, have led many physicians to question the risk/benefit ratio of HDI as adjuvant therapy in melanoma patients at high risk of recurrence [11]. The evaluation of the safety profile of the intensified schedule was therefore one of our main concerns and priorities. Thus, it was also important to learn whether we would be able to administer the intended dose of this intense treatment as planned.

The analysis of toxicity and drug delivery data, on the first 166 patients who entered this study and completed the treatment, shows that we were able to deliver a significantly intensified treatment in the DD/DI arm, without a significant increase in the overall toxicity when compared to the standard HDI therapy.

It is noteworthy that the percentage of patients who received at least 80% of the planned dosage during the induction phase (65%) and the maintenance phase (53%) in HDI arm of this study was similar to those reported in the E1690 and 1684 trials [5,12]. Thus, we were able not only to increase the weekly dose by 17% in the experimental arm, but also to deliver the whole dose intravenously.

Moreover, the overall toxicity observed in the HDI arm of this study was not different from that previously reported by Kirkwood et al [5,12,13].

In the experimental arm, only leukopenia significantly increased (24.4% vs. 8.7%, p = 0.00074) with respect to the HDI arm: this was associated with an increase in neutropenia (44% vs 28%). Despite this higher incidence of leukopenia and neutropenia, neutropenic infections were never recorded and the most often observed infective diseases were primarily skin and cellulite infections localized in the region of the previous lymphadenectomy. This occurrence did not seem to be treatment related, since it was observed in some cases before the start of treatment and, in other cases, even one or two years after its conclusion. Flu-like symptoms and fatigue intensity, experienced by nearly all patients, receded almost completely during the interval-month of DD/DI treatment, although we observed an increase in its frequency and severity in the subsequent cycles. This represented the main reason for an increased dose reduction in the third and fourth cycle in the experimental arm. On the contrary, the patients in the HDI arm tend to develop a better tolerance to fatigue and flu-like symptoms during the course of the treatment as a consequence of an adaptive reaction to the aforementioned specific side effects. An increase in the IFN tolerability in the patients treated with repeated cycles of intravenous high-doses, was observed by Von Wusson P. et al [14], using an interval as long as three months between the cycles. Since, in our study, the interval between the cycles was one month, the subsequent cycles started with incomplete resolution of the previous related effects. Hepatic, gastrointestinal, neurologic, pulmonary, metabolic, and dermatological toxicities are very similar in incidence and severity, in both arms and comparable to those previously reported in the ECOG and Inter-group trials. Even if the dose intensity we were able to deliver in the experimental arm was significantly higher (p = 0.003) than that delivered in HDI arm, the proportion of patients who discontinued the treatment for toxicity or refusal was not significantly higher (26 vs. 36 respectively, p = 0.18). The IFN-induced interstitial pneumonitis we observed in two cases was first described in 1993 in a patient with renal cell carcinoma [15]. Other cases have been described in patients with hepatitis C [16], or chronic myelogenous leukemia [17], although these are the only melanoma cases reported to our knowledge. The incidence of this complication, analyzed in 545 patients affected by chronic hepatitis C, was less than 1% (4 cases) [18], and all patients recovered after IFN withdrawal and corticosteroid therapy. We tried to avoid the use of steroids in our patients with the aim of not perturbing the possible immunologic anti-tumor effects related to IFNα2b: the complete clinical resolution of the interstitial pneumonia observed in our two cases, proved that the use of steroids is not always essential. This severe, even rare, complication, reported also during the pegylated interferon alpha 2b treatment [19], should be taken into account since, in our first case, it was firstly mistaken for metastatic lung lymphangitis.

The main end-point of this study is to investigate whether the increase of dose intensity, and the related increase in IFN concentration, obtained by the exclusive intravenous administration of IFNα2b, could translate into a significantly better 5-year overall survival with respect to the ECOG 1684 schedule. Moreover, an increased benefit combined with the shortening in the treatment duration could offset the acknowledged toxicity of high-dose IFNα. The data derived from this planned analysis on safety and delivered dose demonstrated that our experimental schedule is feasible and gives us sound ground to continue patient accrual in order to meet the primary end-point efficacy.

Since the main toxicities related to the use of high-dose interferon are quickly recovered after its withdrawal, and thanks to the shortening of the whole duration of the treatment by five months (42%), our schedule could have a great positive impact in the risk/benefit ratio of high dose interferon.

## Competing interests

The author(s) declare that they have no competing interests.

## Authors' contributions

VCS conceived the study, participated in its design, carried out chemotherapy, collected the clinical data and drafted the manuscript. AR, MG, AP and RR conceived the study, participated in its design, carried out chemotherapy and collected the clinical data. MDP, EN, BS, MM carried out chemotherapy and collected the clinical data. PDB and GLDS participated in the design, managed the data collection, performed the statistical analysis and drafted the manuscript. All authors read and approved the final manuscript.

## Pre-publication history

The pre-publication history for this paper can be accessed here:



## Figures and Tables

**Table 1:** Patients' demographic and baseline disease characteristics

	**Number of patients (%)**			
**Patients' characteristics**	**DD/DI arm (N = 86)**		**HDI arm (N = 80)**	

**Age, years**				
*Mean (SD)*	47 (13)		52 (13)	
**Gender**				
*Male*	47	(55)	54	(67)
*Female*	39	(45)	26	(33)
**ECOG Performance Status**				
*0*	81	(94)	76	(95)
*1*	5	(6)	4	(5)
**Site of primary tumor**				
*Head/neck*	3	(3)	6	(7)
*Trunk*	50	(58)	40	(50)
*Upper limb*	7	(8)	6	(8)
*Lower limb*	26	(30)	24	(30)
*Unknown*	0	(0)	4	(5)
**Ulceration**				
*No*	45	(52)	36	(45)
*Yes*	33	(39)	34	(43)
*Unknown*	8	(9)	10	(12)
**Breslow's thickness (mm)**				
≤ *1*	2	(2)	7	(9)
*1.01 – 2.0*	27	(31)	18	(23)
*2.01 – 4.0*	30	(35)	29	(36)
*>4*	22	(26)	17	(21)
*Unknown*	5	(6)	9	(11)
**Nodal classification**				
*N0*	0	(0)	2	(2)
*N1*	56	(65)	46	(58)
*N2*	19	(22)	24	(30)
*N3*	11	(13)	8	(10)

**Table 2:** Adverse events (all grades) by treatment arm

	**Number of patients (%)**															
	**DD/DI arm (N = 86)**								**HDI arm (N = 80)**							

**Event**	***Grade 1***		***Grade 2***		***Grade 3***		***Grade 4***		***Grade 1***		***Grade 2***		***Grade 3***		***Grade 4***	

**Flu-like symptoms**																
*Fatigue*	19	(22)	26	(30)	25	(29)	0		14	(17)	27	(34)	19	(24)	3	(4)
*Fever*	28	(33)	33	(38)	7	(8)	0		25	(31)	23	(29)	8	(10)	1	(1)
*Rigors/chills*	15	(17)	5	(6)	0		0		12	(15)	3	(4)	3	(4)	0	
*Arthralgia/myalgia*	26	(30)	17	(20)	1	(1)	0		29	(36)	12	(15)	3	(4)	1	(1)
*Sweating*	8	(9)	3	(3)	0		0		8	(10)	1	(1)	1	(1)	0	
**Gastrointestinal**																
*Nausea/vomiting*	32	(37)	12	(14)	2	(2)	0		27	(34)	12	(15)	1	(1)	0	
*Constipation*	13	(15)	5	(6)	0		0		9	(11)	3	(4)	0		0	
*Diarrhea*	11	(13)	1	(1)	0		0		5	(6)	1	(1)	1	(1)	1	(1)
*Anorexia*	30	(35)	10	(12)	3	(3)	0		14	(17)	12	(15)	6	(7)	0	
**Hematological**																
*Leukopenia*	13	(15)	32	(37)	21	(24)	0		28	(35)	25	(31)	7	(9)	0	
*Anemia*	12	(14)	0		1	(1)	0		16	(20)	3	(4)	0		0	
*Granulocytopenia*	7	(8)	21	(24)	31	(36)	7	(8)	13	(16)	23	(29)	21	(26)	2	(2)
*Platelets*	11	(13)	1	(1)	3	(3)	1	(1)	16	(20)	5	(6)	0		0	
*Hemorrhage*	2	(2)	4	(5)	0		0		1	(1)	1	(1)	0		0	
**Hepatic function**																
*Bilirubin*	3	(3)	2	(2)	0		0		6	(7)	0		0		0	
*SGOT/SGPT*	16	(19)	11	(13)	18	(21)	15	(17)	21	(26)	24	(30)	18	(22)	3	(4)
*Alkaline phosphatase*	8	(9)	2	(2)	0		0		8	(10)	3	(4)	0		0	
*Liver dysfunction*	0		2	(2)	1	(1)	0		3	(4)	1	(1)	1	(1)	0	
**Neurologic**																
*Depression/anxiety*	19	(22)	14	(16)	7	(8)	0		20	(25)	12	(15)	12	(15)	1	(1)
*Cognitive disturbance*	10	(12)	10	(12)	4	(5)	0		10	(12)	7	(9)	2	(2)	0	
**Cardiovascular**																
*Hypertension*	6	(7)	1	(1)	0		0		2	(2)	1	(1)	2	(2)	0	
*Hypotension*	1	(1)	1	(1)	0		0		5	(6)	4	(5)	0		0	
*Ritmo*	0		2	(2)	0		0		1	(1)	1	(1)	0		0	
*Cardiac dysfunction*	6	(7)	4	(5)	0		0		7	(9)	6	(7)	2	(2)	1	(1)
**Pulmonary**																
*Dyspnea*	8	(9)	3	(3)	0		0		13	(16)	4	(5)	0		0	
*Cough*	7	(8)	1	(1)	0		0		13	(16)	1	(1)	0		0	
**Dermatology**																
*Skin*	15	(17)	7	(8)	4	(5)	0		15	(19)	11	(14)	2	(2)	1	(1)
*Local*	1	(1)	0		1	(1)	0		5	(6)	1	(1)	1	(1)	0	
**Metabolic**																
*Hyperglycaemia*	7	(8)	3	(3)	3	(3)	0		5	(6)	3	(4)	0		0	
*Hypoglycaemia*	1	(1)	2	(2)	4	(5)	2	(2)	1	(1)	3	(4)	0		1	(1)

**Table 3:** Treatment compliance

	**Number of cycles (%)**			
	**DD/DI arm**		**HDI arm**	

N° cycles delivered	272		701	
According to protocol	72	(26)	187	(27)
Modified in dose less than 20%	64	(23)	176	(25)
Modified in dose more than 20%	136	(50)	338	(48)

**Table 4:** Number of patients who completed treatment and reasons for discontinuation

	**Number of patients (%)**			
	**DD/DI arm (N = 86)**		**HDI arm(N = 80)**	

Completed therapy	54	(63)	32	(40)
Reasons for discontinuation				
*Toxicity*	11	(13)	14	(17)
*Refusal*	11	(13)	15	(19)
*Disease Progression*	10	(12)	19	(24)

**Table 5:** Proportion of patients who received more than 80% of the target dose

	**Number of patients (%)**			
**Time from randomization**	**DD/DI arm (N = 86)**		**HDI arm(N = 80)**	

Induction	56	(65)	52	(65)
3 months	35	(49)	42	(64)
5 months	25	(42)	33	(55)
7 months	20	(36)	23	(43)

**Table 6:** Dose of interferon received and actual dose intensity.

	**DD/DI arm (N = 86)**		**HDI arm (N = 80)**	
	**Mean (SD)**	**Median (Range)**	**Mean (SD)**	**Median (Range)**

Target dose (MU/m^2^)	1600		1840	
MU/m^2 ^received	974.6 (455.6)	1034.4 (40 -1620.2)	1052.2 (509.2)	1155.9 (20-1888.9)
Target DI* MU/m^2^/week	50		35.4	
Actual DI MU/m^2^/week	37.1 (10.8)	36.4 (5–52.1)	34.1 (14.4)	30.7 (5–91.8)
% of target DI	0.74 (0.2)	0.73 (0.1–1.0)	0.96 (0.4)	0.87 (0.1–2.6)

*DI: Dose Intensity

**Table 7:** Supportive therapy

	**Number of patients (%)**			
**Cycle 1 (intravenously)**	**DD/DI arm (N = 86)**		**HDI arm (N = 80)**	

Paracetamol	79	(92)	71	(89)
Anti-emetics	47	(55)	44	(55)
Anti-diarrhea	3	(3)	2	(2)
Anti-anxiety	20	(23)	15	(19)
Vitamin	22	(26)	15	(19)
**Subsequent cycles**	**DD/DI arm (N = 71)**		**HDI arm (N = 69)**	
Paracetamol	64	(90)	55	(80)
Anti-emetics	44	(62)	21	(30)
Anti-diarrhea	6	(8)	2	(3)
Anti-anxiety	19	(27)	16	(23)
Vitamin	21	(30)	18	(26)
